# *Escherichia coli* mastitis strains: *In vitro* phenotypes and severity of infection *in vivo*

**DOI:** 10.1371/journal.pone.0178285

**Published:** 2017-07-20

**Authors:** Perrine Roussel, Adeline Porcherie, Maryline Répérant-Ferter, Patricia Cunha, Christophe Gitton, Pascal Rainard, Pierre Germon

**Affiliations:** ISP, INRA, Université François Rabelais de Tours, UMR 1282, Nouzilly, France; University of Illinois, UNITED STATES

## Abstract

Mastitis remains a major infection of dairy cows and an important issue for dairy farmers and the dairy industry, in particular infections due to *Escherichia coli* strains. So far, properties specific to *E*. *coli* causing mastitis remain ill defined. In an attempt to better understand the properties required for *E*. *coli* to trigger mastitis, we used a range of *in vitro* assays to phenotypically characterize four *E*. *coli* strains, including the prototypical *E*. *coli* mastitis strain P4, possessing different relative abilities to cause mastitis in a mouse model. Our results indicate that a certain level of serum resistance might be required for colonization of the mammary gland. Resistance to neutrophil killing is also likely to contribute to a slower clearance of bacteria and higher chances to colonize the udder. In addition, we show that the four different strains do induce a pro-inflammatory response by mammary epithelial cells but with different intensities. Interestingly, the prototypical mastitis strain P4 actually induces the less intense response while it is responsible for the most severe infections *in vivo*. Altogether, our results suggest that different strategies can be used by *E*. *coli* strains to colonize the mammary gland and cause mastitis.

## Introduction

In dairy cattle worldwide, mastitis remains a major issue. Clinical forms of mastitis are characterized by the inflammation of the udder, thus impairing mammary cell functions. As a consequence, it constitutes a threat for the health of cows. In addition, considering on the one hand the treatment costs to cure the disease, and on the other hand a shortfall in milk production, mastitis is also a major source of financial losses for the dairy farmers.

Mastitis results in most cases from the invasion of the udder by bacteria. Among responsible pathogens, *Escherichia coli* is of importance because of its prevalence and the damages this bacterium may induce. To the least, *E*. *coli* reduces milk production yields, at most it can cause severe dysfunction of the udder and can sometimes lead to the death of the animal [1].

The current dogma is that the severity of *E*. *coli* mastitis is mainly due to the host predispositions, in particular its health status, stage of lactation, parity and genetic background [2]. Indeed, only a few distinctive features of mastitis *E*. *coli* isolates have been unravelled. An increased proportion of mastitis isolates belong to phylogroup A compared to other *E*. *coli* strains [3]. Studies have shown a low prevalence in mastitis *E*. *coli* strains of different virulence determinants described in other *E*. *coli* pathovars, such as toxins, adhesins and invasins, secretion machineries or iron acquisition systems [4–8]. Mastitis *E*. *coli* strains are thus rather characterized by the lack of virulence genes than by the presence of a combination of virulence genes [8, 9].

Regarding phenotypic characteristics of mastitis *E*. *coli* strains, the literature reports a number of potentially important phenotypes. The ability of bacteria to grow in milk seems to contribute to the colonisation of the udder: strains with the lowest growth in whole milk are found among fecal isolates but not isolates from mastitis cases [10] while ability to grow in mastitic milk is reduced in fecal isolates [5]. Interestingly, *E*. *coli* strains isolated from persistent mastitis cases showed increased adherence and persistence in a mammary epithelial cell line [4]. Yet, the genetic bases for these phenotypes remain unknown and their *in vivo* relevance needs clarification [11, 12]. Because neutrophils are essential for the clearance of bacteria, an increased resistance to phagocytosis and killing by neutrophils is also likely to aggravate the infection [13–17].

Initiating the innate response that leads to this neutrophil recruitment is also a key event in eliminating the invading pathogen. Mammary epithelial cells have been shown to play a significant role in this response triggered upon recognition of bacterial motifs called MAMPs (Microbial-Associated Molecular Patterns), which can be recognized by specific and highly conserved Patterns Recognition Receptors (PRRs) [18]. The contribution of MEC to the host’s response seems to be of the utmost importance as recently described [19]. Yet, although the pro-inflammatory response of MEC to purified compounds or to some bacterial strains or species has been investigated by us and others, the diversity of *E*. *coli* strains in terms of their capacity to trigger the innate immune system has not been thoroughly examined. The major MAMP implied in *E*. *coli* pathogenicity in mastitis has been shown to be the lipopolysaccharide (LPS), which covers the outer membrane of the bacterial wall [2]. LPS is recognized by soluble or membrane CD14, associated with LBP (LPS-binding protein), which leads the LPS to a toll-like receptor (TLR), TLR4. Other MAMPs include lipoproteins, recognized by TLR2, associated with TLR1 or TLR6, as well as peptidoglycans, recognized by cytoplasmic receptors NOD1 and NOD2 [20]. One could therefore hypothesize that a delay in the recognition of the pathogen induces a delayed response, promotes the multiplication of the pathogen and leads to more severe infections [13].

This study aimed at characterizing different phenotypic characteristics and compared them with the severity of mastitis caused by different *E*. *coli* strains in a mouse model. Based on multiple references in the literature, the murine model was used as a surrogate to measure the degree of severity of mastitis induced by different strains. Earlier studies have highlighted the value of this model in the study of the inflammatory response triggered by intra-mammary infection by *E*. *coli* [16, 17, 21, 22] Indeed, murine mastitis recapitulates the most relevant characteristics of bovine mastitis: it is characterized by the production of pro-inflammatory mediators in milk; then, a strong recruitment of neutrophils occurs which allows the clearance of bacteria [16, 17, 21, 22]. In addition, good similarities between results obtained in mice and cows were reported [11].

In particular, among the multiple *in vitro* phenotypes analysed, this study unravelled that different *E*. *coli* strains had different abilities to trigger the innate immune system.

To summarize, the ability of a strain to cause mastitis is likely to rely on a combination of properties that allow growth in milk, resistance to host defences, stimulation of the innate immune system and possibly adhesion to epithelia.

## Material and methods

### Ethic statement

Animal experiments were conducted at the Experimental Infectiology Unit (INRA Val de Loire) with the approval of the ethics committee « Comité d’éthique pour l’expérimentation animale du Val de Loire » (agreements No 2012-10-12 and 2012-11-2). Animal studies were compliant with all applicable provisions established by the European directive 2010/63/UE.

### *E*. *coli* strains

*E*. *coli* strains used in this study and their relevant phenotypes are listed in Table 1. Strain P4 is a prototypical mastitis strain isolated from a case of clinical mastitis [23]. Strains D6-117.07 and D6-117.29 were isolated from cases of severe bovine clinical mastitis defined by the presence of local inflammatory signs (inflamed udder) and systemic symptoms (fever above 39.6°C, prostration, loss of appetite and rumen contraction). Strain B1171 strain was kindly provided by James Leigh (Nottingham University, United Kingdom) and was chosen based on its phenotype for resistance to neutrophil killing. Strain *E*. *coli* K-12 MG1655 was included in the study to constitute a negative control as it is considered a non-pathogenic *E*. *coli* strain and was obtained from the *E*. *coli* Genetic Stock Center (CGSC#6300). Strains serotyping was performed at the Laboratorio de Referencia de *Escherichia coli* (Spain). Strains are stored at -80°C, in BHI (Brain Heart Infusion) medium containing 15% glycerol.

**Table 1:** List of strains used in this study.

Strains	Type/Origin	Phylogroup ^(a)^	Serotype	Reference
*E*. *coli* P4	Clinical mastitis	A (ST10)	O32:H37	[8]
*E*. *coli* K-12 MG1655	Commensal	A (ST10)	OR:H48	[24]
*E*. *coli* B1171	Unknown	A (ST1672)	O141:H11	this study
*E*. *coli* D6-117.07	Clinical mastitis	A (ST10)	O45:H27	[9]
*E*. *coli* D6-117.29	Clinical mastitis	A (ST10)	O42:H37	this study

^(a)^ sequence types (ST) were determined using the MLST scheme of EnteroBase (http://enterobase.warwick.ac.uk)

### Murine mastitis model

*E*. *coli* strains were first seeded in 10 mL BHI medium for 8 h at 37°C without agitation. The precultured bacterial suspensions were then diluted to 1/100 in BHI medium and incubated for 16 h at 37°C without agitation. Bacterial cultures were centrifuged (15 min at 600 x *g*) and washed with sterile 0.9% NaCl (Cooper). Bacterial concentration was determined by optical density measurement at 600 nm. The bacterial inoculum was adjusted to 2.10^4^ colony forming units (cfu)/mL in 0.9%NaCl.

C57BL/6J pregnant mice were obtained from Janvier breeder, and raised at the Experimental Infectiology Unit (INRA Val de Loire). Experiments were done with mice at their 8th day of lactation, with 5 to 6 suckling pups. Suckling pups were disposed of 4 h before each experiment. The mice received intraperitoneally 250 μL of 0.9% NaCl, 15% ketamin (Imalgene 1000), 5% xylazine (Rompun). Each mouse received 50 μL of bacterial inoculum (10^3^ cfu/gland) in the 4th right mammary gland and 50 μL of sterile 0.9% NaCl in the 4th left mammary gland. The infections were done through the teat canal using a 33G blunt-end needle (World Precision Instruments). Mice were sacrificed 24 or 48 h.p.i by cervical dislocation by trained personnel. Lesions were scored as 0 (no lameness; normal size, consistency and vasculature of the udder; presence of milk with normal aspect in both infused mammary glands) to 4 (loss of motility; induration and general inflammation of the udder). The mammary glands were then isolated under sterile conditions, and transferred in DPBS (without Ca and Mg).

Each mammary gland was weighed, and minced. On a part of the minced glands, the bacterial loads were determined after 10-fold serial dilutions in sterile PBS and plating on TSA plates.

The remaining supernatant was digested by collagenase III (4 200 U/g of tissue; Worthington) in RPMI 1640 for 30 min at 37°C, under brisk shake. The cell pellet was separated by concentration gradients in Percoll (GE Healthcare) at 40 and 80%. The interface corresponding to the leukocytes was collected, and washed by DPBS, 5% SVF, and resuspended in FACS buffer (DPBS, 2% of goat serum (Gibco), 2 mM EDTA). Cells were counted by Trypan blue staining (Gibco). The cells were incubated with Fc block buffer (anti-CD13 and anti-CD32 diluted to 1/100 in FACS buffer) for 20 min at 4°C in the dark. Cells were then incubated in presence of antibodies anti-CD45-APC and anti-Ly6G-PE (BioLegend), and finally fixed during 20 min 4°C in the dark in FACS Lysing Solution (BD Biosciences), and conserved at 4°C in the dark. Acquisitions were done using a FACSCalibur cell analyzer (BD Biosciences) and results were analyzed using FlowJo (TreeStar Inc.) software to quantify neutrophils (CD45+ Ly6G+ cells).

On the other part of the minced glands, anti-proteases (Sigma-Aldrich) were added and cytokine quantifications were done by ELISA (Duoset assays, R&D Systems) as described [25].

Each series of experiments were done on 4 to 8 mice per time point.

### Growth in bovine milk

Fresh milk was obtained from the Experimental Unit of Animal Physiology (UE PAO, INRA Val de Loire). Samplings were performed under conditions as aseptic as possible on two primiparous cows between their 5th and 8th week of lactation, without any reported history of mastitis, and with somatic cell count (SSC) below 50 000 c/mL. Absence of infection in the sampled quarter was verified the day before and the day of each sampling by plating 100 μL of milk on blood agar. SSC were obtained using a Fossomatic 90 apparatus (A/S N. Foss, Denmark). The fresh whole milk was used untreated. Commercial microfiltered organic milk came from « La Marguerite » farm (Monnaie, France) and was stored at 4°C until use.

We first ensured that there was no aggregation of the different strains in milk (data not shown). *E*. *coli* strains were first seeded in DMEM-F12 (Gibco) medium for 8 h at 37°C without agitation. The precultured bacterial suspensions were then diluted 100-fold in DMEM-F12 medium and incubated for 16 h at 37°C without agitation. Bacterial cultures were then centrifuged (15 min at 600 x *g*) and washed with sterile HBSS. The bacteria concentration was determined by optic density measurement at 600 nm. To analyze growth in milk, 10 mL of milk were seeded with the bacterial suspension at a concentration of 10^4^ cfu/mL. The inoculated milk was immediately dispensed in 1.5 mL microtubes (1.5 mL per tube). Microtubes were incubated at 37°C without agitation. Every hour, CFUs from one microtube were counted by serial dilutions and plating on TSA plates and Doubling times were calculated.

### Resistance to bovine serum

Serum was prepared from blood collected from 9 healthy dairy cows in commercially available 10 mL evacuated non-coated tubes (Venosafe^™^, Terumo^®^ Europe). After coagulation, sera were collected, checked for sterility, pooled and stored at -80°C in 1mL aliquots. *E*. *coli* strains were first seeded in DMEM-F12 (Gibco) medium for 8 h at 37°C. The precultured bacterial suspensions were then diluted 100-fold in DMEM-F12 medium and incubated for 16 h at 37°C. Before each experiment, bacterial cultures were centrifuged (15 min at 600 x *g*) and washed by sterile HBSS. The bacteria concentration was determined by optic density measurement at 600 nm. To analyze resistance to bovine serum, bacterial cultures were seeded (10^4^ cfu/mL) in DPBS Ca2+, Mg2+ (Dulbecco’s Phosphate Buffer Saline with Ca and Mg, Lonza), containing cow serum at different concentrations of serum (5, 10, 20, 40 or 50%). Heat inactivated serum (56°C—30 min) was used as a control. Bacterial suspensions were incubated for 3 h at 37°C. At the end of the incubation, since strain D6-117.29 was found to aggregate in bovine serum, the samples of this strain were sonicated (7 s, power 2, voltage 3; Vibra Cell sonicator; Sonics & Material, Connecticut). The bacterial loads were determined after dilutions of bacterial suspensions in sterile PBS and plating on TSA agar plates. Survival percentages at one serum concentration were determined by dividing the number of cells obtained after 3h in serum by the number of cells obtained after 3h in heat inactivated serum. Resistance tests to bovine serum were repeated three times.

### Bovine mammary epithelial cells

PS cells, a recently described mammary epithelial cell line, were cultured as previously described [26] in Advanced DMEM/HF-12 medium (Gibco) containing 4 ng/mL of hydrocortisone (Gibco), 2 mM of glutamine (Gibco) and 20 mM of HEPES (Biowhittaker), IGF-I (Insulin-like Growth Factor; 10 ng/mL; Peprotech), FGF (Fibroblast Growth Factor; 5 ng/mL; Peprotech) and EGF (Epidermal Growth Factor; 5 ng/mL; Sigma).

### Adhesion and invasion of MECs by *E*. *coli*

To evaluate the ability of *E*. *coli* to adhere to or to invade the PS cell line, the PS cells were infected by the different strains for 3 h at an MOI of 10. Incubation were performed either in stimulation medium [26] or fresh milk. At 3 h.p.i., the supernatants were collected and the bacterial loads, representing bacteria free in the medium (NUM), were determined after dilutions of bacterial suspensions in sterile PBS and numeration on TSA plates. Cells were then washed twice by DPBS and lysed by addition of 0.2% Triton X-100 diluted in DPBS and incubated at 37°C for 10 min. The bacterial loads, representing adherent and invasive bacteria (ADH), were determined after dilutions of bacterial suspensions in sterile PBS and numeration on TSA. To determine the percentage of invasive bacteria, PS cells were washed three times by DPBS and medium containing 100 μg/mL of gentamicin was added for 30 min at 37°C. After 30 min, cells were washed by DPBS, lysed by 0.2% de Triton X-100 diluted in DPBS and incubated at 37°C for 10 min. The bacterial loads, representing invasive bacteria (INV), were determined after dilutions of bacterial suspensions in sterile PBS and numeration on TSA. Percentages of adhesion and invasion were determined by calculating the ratios (ADH-INV)/(NUM+ADH)*100 and INV/(NUM+ADH)*100 respectively.

### Stimulation of cells with live bacteria or purified bacterial agonists

Stimulation assays were performed as described previously [26]. CXCL8 production by PS cells was quantified by sandwich ELISA with reference to standard as described previously [27]. Response was also analysed by quantitative real-time PCR as described before [28]. Recombinant bovine CD14 was purified from S2 cells transfected with plasmid pCG11. The CD14 cDNA was obtained by RT-PCR from RNA of bovine blood monocytes using primers GTTGAGATCTGACACAACAGAACCCTGCGA and AGTATTCGAACGCGAAGCCTCGGGCTCCTT and cloned downstream of a BiP signal sequence and upstream of a 6-His tag in the BglII/BstBI sites of plasmid pMT-puro [29]. This plasmid was then transfected in S2 cells grown in InsectExpress medium (Lonza) without serum. After puromycin selection (1μg/ml) of transfected cells, expression of CD14 was induced by 500μM CuSo4. Purification from 1L of supernatant 6 days after induction was achieved by affinity chromatography on Ni-NTA resin (Qiagen). Purification was verified by SDS-PAGE and, after silver staining of gels, purity was estimated to be >99%. Sequence of the purified rbCD14 was confirmed by mass-spectrometry and comparison to the bovine CD14 sequence (139 peptides obtained yielding a coverage of 89%). No significant match to *Drosophila melanogaster* database was obtained.

### Stimulation of HEK-TLR2 and HEK-TLR4 cells

HEK cell lines transfected with hTLR2 or hTLR4 (InvivoGen) were used. These cells were cultivated in DMEM 4.5 μg/mL glucose (Gibco) containing 2 mM of glutamine, 20 mM of HEPES, 10% heat-inactivated FCS and 1x HEK-Blue Selection medium (InvivoGen). HEK cells were incubated for 3h at an MOI of 1 with live *E*. *coli* grown as previously described [26]. Bacteria were removed, cells were washed once with HBSS and stimulation medium containing gentamicin (100 μg/mL) was then added. Incubation was extended for 5h, then supernatants were collected.

### *Escherichia coli* survival to exposure to bovine PMNs

Bovine PMNs were isolated in conditions minimizing their activation and with a maximum of aseptic conditions. Blood samples were collected from cows in Venosafe EDTA tubes (Venosafe^™^, Terumo^®^ Europe) and centrifuged at 1300 x *g* for 20 min at 20°C. Two milliliters of the pellet containing the granulocytes were transferred in 5 mL of Red Blood lysing solution (Sigma) for 3 min at room temperature. The cells were then washed with DPBS (no calcium, no magnesium—Gibco), and centrifuged for 5 min at 200 *g*, 20°C. The cell pellet was then resuspended in DPBS and centrifuged 5 min at 200 *g*, 20°C. The cell pellet was then resuspended in DPBS-AH (DPBS Ca^2+^, Mg^2+^; 10 mM HEPES; 0.1% bovine serum albumin (weigh/volume) (Sigma)). After numeration on a Malassez slide, the cell concentration was then adjusted to 2.10^6^ /mL. *E*. *coli* strains were first seeded in BHI medium for 8 h at 37°C without agitation. The precultured bacterial suspensions was then diluted 100-fold in BHI medium and incubated for 16 h at 37°C without agitation. Before each experiment, bacterial cultures were centrifuged (15 min at 600 *g*) and washed with sterile DPBS. Bacteria concentrations were determined by optic density measurement at 600 nm, and adjusted to 2.10^7^ cfu/mL in DPBS-AH– 10% heat inactivated serum. Five hundred microliters of the bacteria suspension were placed in a 5 mL Falcon tube. After 15 minutes at 37°C without agitation, PMNs were then added to bacteria in order to achieve a MOI of 10. A negative control was prepared using bacteria in the presence of heat-inactivated serum only. The suspensions were then incubated under slow rotation for 1 h at 37°C.

At the end of the incubation, the suspensions were diluted 10-fold in 0.2% of Triton X-100 and bacterial loads were determined after further dilutions in sterile PBS and plating on TSA agar plates. The percentage of bacterial survival was obtained after dividing the number of bacteria counted after 1h in the presence of neutrophils by the number of bacteria counted after 1h in the absence of neutrophils.

### Statistical analysis

Data were first compared using the Kruskal and Wallis test followed by exact permutation tests using StatXact software. A p-value < 0.05 was considered significant.

## Results

### Mastitis induced by *E*. *coli* in mice

Strains tested included the non-pathogenic *E*. *coli* K-12 substr. MG1655, the prototypical mastitis isolate strain P4, one *E*. *coli* strain isolated from a case of acute clinical mastitis, D6-117.29, and strain B1171 selected from our collection based on its resistance to neutrophil phagocytosis (see below).

**Fig 1:**
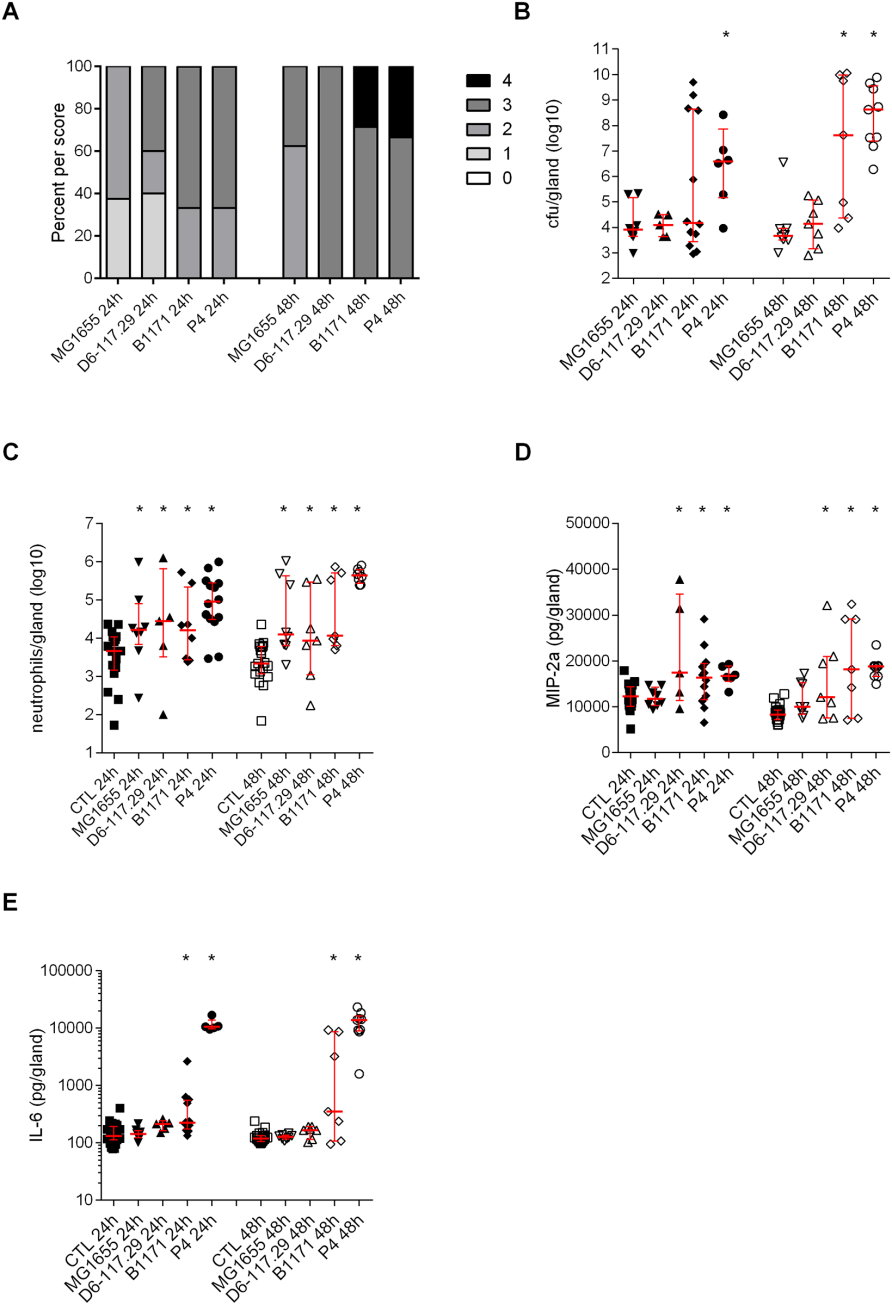
Severity of infection induced by mastitis *E*. *coli* strains in mice after intra-mammary inoculation. Mice were inoculated with 10^3^ cfu per gland in the right 4th mammary gland and with an equal volume of PBS in the left 4th mammary gland (control gland, CTL). A, percentages of mice by clinical scores. B, cfu were counted 24 and 48 h post-inoculation in the infected mammary gland. C, neutrophil recruitment in the infected glands and control glands was measured by FACS of mammary cells labelled with anti-CD45 and anti-Ly6G. D and E, mediators of inflammation were quantified by ELISA (D, MIP-2a; E, IL-6). Data presented are values from each individual animal with medians and interquartile ranges indicated as vertical bars. * indicates statistical significance (P < 0.05). P-values were calculated using exact permutation tests after global comparison using a Kruskal and Wallis test. A: comparison against MG1655; B, C, D: comparison against 4th right mock-infected gland.

The inoculum corresponded to 10^3^ cfu/gland. All mock infected glands appeared free of any infection. Mean bacterial concentrations remained low in mice infected by MG1655 and D6-117.29 (median of 8.06 x 10^3^ and 2.31 x 10^4^ cfu/gland, respectively–Fig 1A). Bacterial concentration did not vary for D6-117.29 at 48 h.p.i. (2.7 x 10^4^ cfu/gland), but decreased for MG1655 (4.15 x 10^3^ cfu/gland). On the contrary, bacterial counts in the mammary gland at both time points by P4 was significantly higher (3.81 x 10^6^ and 4.14 x 10^8^ respectively) than colonization by MG1655. Interestingly, at both time points, almost half of the mice infected with strain B1171 had high cfu/gland (above 10^8^ cfu/gland) while the others had lower counts (approx. 10^4^ cfu/gland).

In all infected glands, significant numbers of neutrophils were recruited compared to mock inoculated glands (Fig 1B).

Because the proinflammatory cytokine IL-6 and the chemokine MIP-2 contribute to the inflammatory reaction of the host, their production in infected glands was analyzed. MIP-2 production was significantly increased in mice infected with strains P4, B1171 and D6-117.29 (Fig 1C). Significant production of IL-6 was observed in mice infected with strains P4 and B1171 compared to mock-infected glands, the highest levels being reached with P4 (Fig 1D).

Taking into account these different parameters, the strains can tentatively be ranked in terms of infection severity: P4 induces the most severe infections while strain MG1655 triggers the least severe infections. Strain D6-117.29 also induces non severe infections (low bacterial load) yet with slightly higher pro-inflammatory response. Strain B1171 is intermediate between P4 and D6-117.29.

### Growth of *E*. *coli* in fresh milk

**Fig 2:**
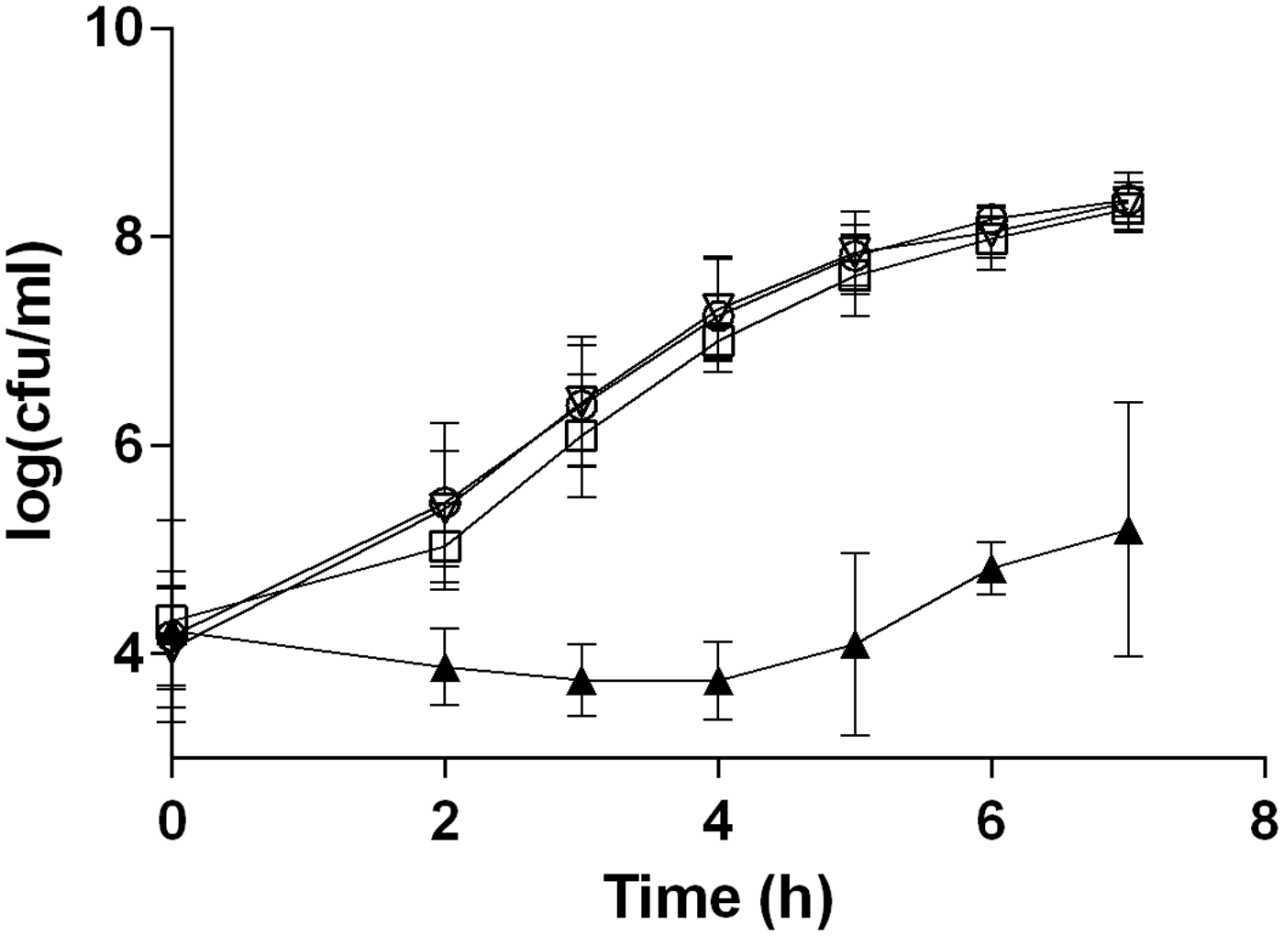
Growth in milk of selected *E*. *coli* strains. Fresh whole milk was inoculated with 10^4^ cfu/mL of strains MG1655 (▲), D6-117.29 (▽), B1171 (○) and P4 (◻). Inoculated milk was distributed in 1.5 mL microtubes (1.5 mL per tube) and bacteria from one tube were counted at each time point by 10-fold serial dilution and plating on TSA agar plates. Data presented are means of cfu/ml calculated from three independent experiments.

Because the potential of *E*. *coli* to colonize the udder depends partially on its ability to multiply in milk, growth of the different *E*. *coli* strains was therefore evaluated in fresh whole milk. In fresh milk, strain MG1655 did not grow at all while the other three strains showed similar growth (Fig 2). Doubling times for strains D6-117.29, B1171 and P4 in fresh milk were 18.6 ±1.6 min, 19.1 ±2.8 min and 17.3 ±2.2 min, respectively, and were not statistically different. In commercial milk, all four strains grew with doubling times not significantly different and varying between 17 and 19 minutes (data not shown).

### *E*. *coli* survival in serum

**Fig 3:**
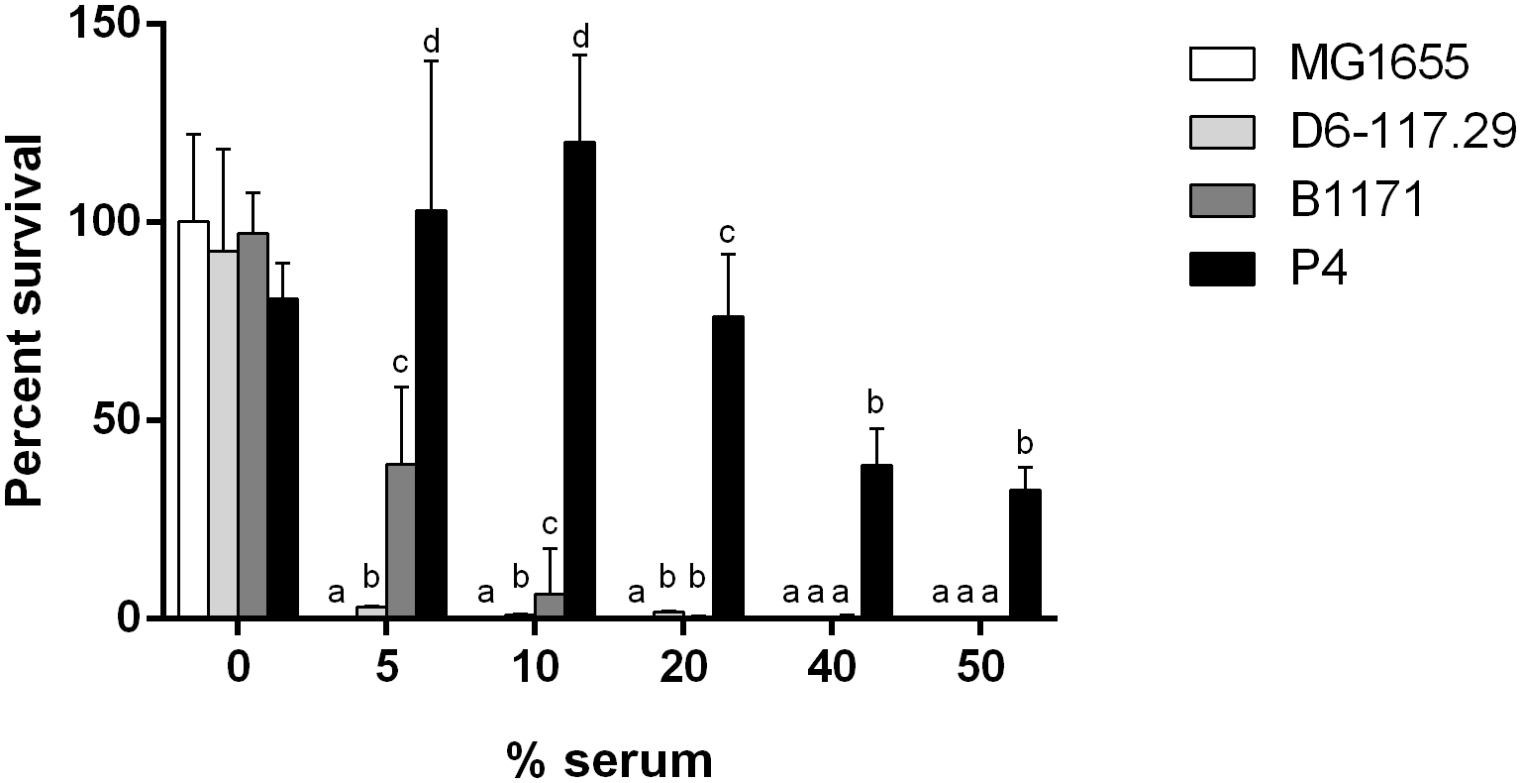
Resistance of *E*. *coli* strains to bovine serum. Strains were incubated, with a starting inoculum of 10^4^ cfu/mL, in the presence of whole bovine serum or heat-inactivated serum at concentrations ranging from 0% to 50% for 3 hours. Data presented are means and standard deviations of the percentage of survival expressed as 100*(number of bacteria obtained after 3h in x % serum / number of bacteria obtained after 3h in x % heat inactivated serum) of at least three independent experiments. Comparison of strains should only be performed taking into account survival values within one concentration group. For each concentration of serum, values with different letters are statistically different (P < 0.05). Statistical difference was calculated independently for each concentration of serum using exact permutation tests after global comparison using a Kruskal and Wallis test.

Milk contains significant amounts of complement that could impede the growth of colonizing *E*. *coli* [30]. Although the role that resistance to serum may play in mastitis is questioned [1], we deemed important to measure precisely, with a low initial inoculum, the resistance of the four strains to bovine serum. The impact of serum was thus tested after 3 h of incubation in the presence of heat-inactivated serum or non-heated serum at concentrations ranging from 5 to 50% (Fig 3). Whatever the concentration, MG1655 was unable to survive in the presence of non-heated serum. Strains D6-117.29 and B1171 were slightly resistant to serum while strain P4 retained 32% viability in the presence of 50% serum.

### Interactions between *E*. *coli* and mammary epithelial cells

When *E*. *coli* enters the udder, and after having multiplied in milk, the bacteria are in contact with host cells, in particular mammary epithelial cells (MEC).

We therefore took advantage of a recently described mammary epithelial cell line to analyze potential differences between strains in terms of ability to adhere to, to invade and to stimulate MECs [26]. Of note, this cell line is able to grow without serum.

**Fig 4:**
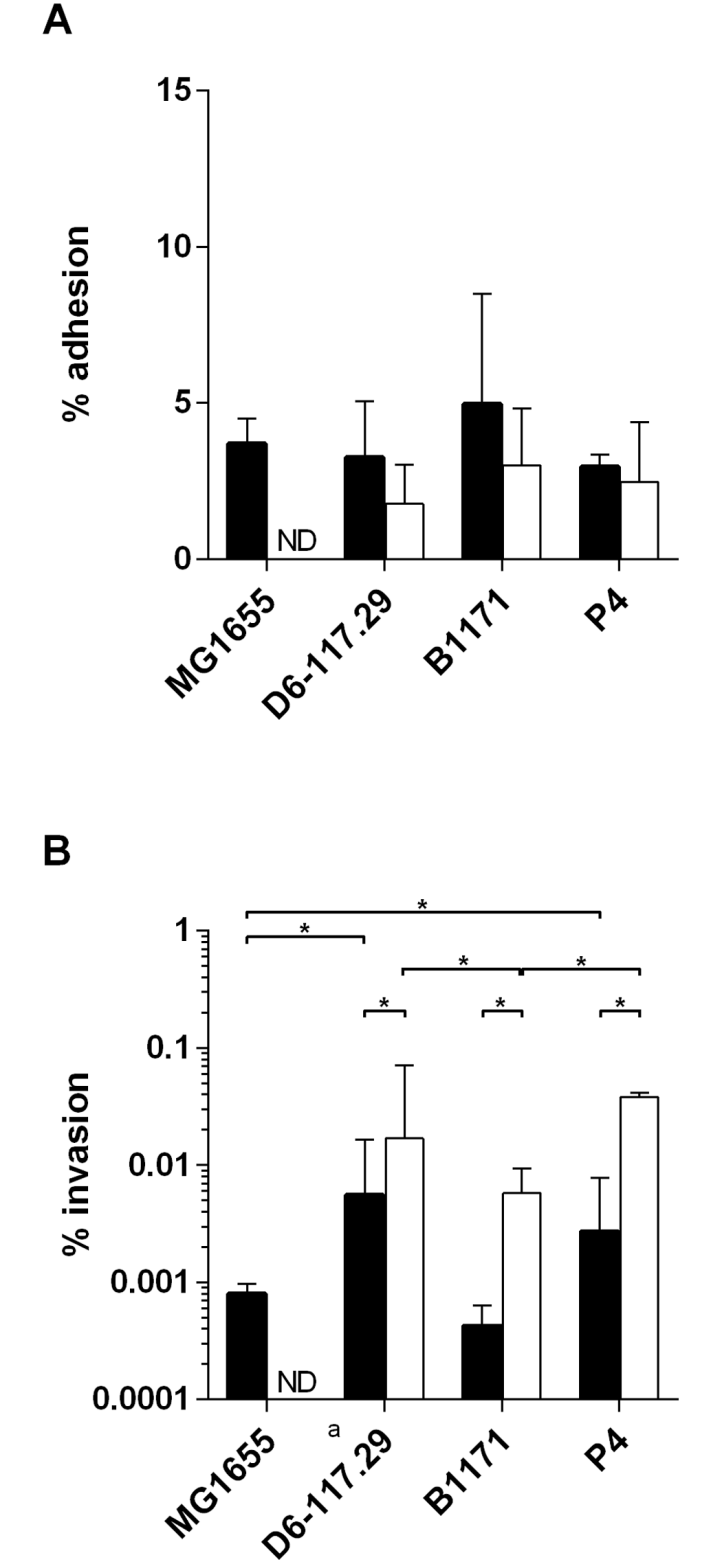
Adhesion and invasion properties of *E*. *coli* strains. PS cells were incubated for 3 h with the indicated strains at a MOI of 10 in stimulation medium (black bars) or fresh milk (white bars). Cells were then washed twice with HBSS. A) cells were lysed by the addition of 0.2% Triton X-100 and bacteria were counted in the supernatant. B) Growth medium with gentamicin was added for 30 minutes, cells were then washed with DPBS, lysed by the addition of 0.2% Triton X-100 and bacteria were counted in the supernatant. Data presented are mean values and SD obtained from 3 independent experiments performed in duplicates. * indicates statistical significance (P < 0.05). p-values were calculated using exact permutation tests after global comparison using a Kruskal and Wallis test.

We first characterized the ability of strains to adhere to and to invade PS cells in culture medium and in fresh milk. In culture medium, results showed that percentages of adhesion were not statistically different between strains. All four strains showed weak invasive properties in culture medium with strains P4 and D6-117.29 showing slightly higher invasion rates (Fig 4A and 4B). In fresh milk, adhesion and invasion could not be quantified for strain MG1655, potentially due to its sensitivity to complement. For the other three strains, the adhesion rates were not statistically different in milk while strains P4 and D6-117.29 were slightly more invasive than strain B1171 (Fig 4A and 4B). It is worth mentioning that, although these three strains showed lower adhesion rates in milk than in culture medium, the invasion rates in milk were higher than in culture medium.

**Fig 5:**
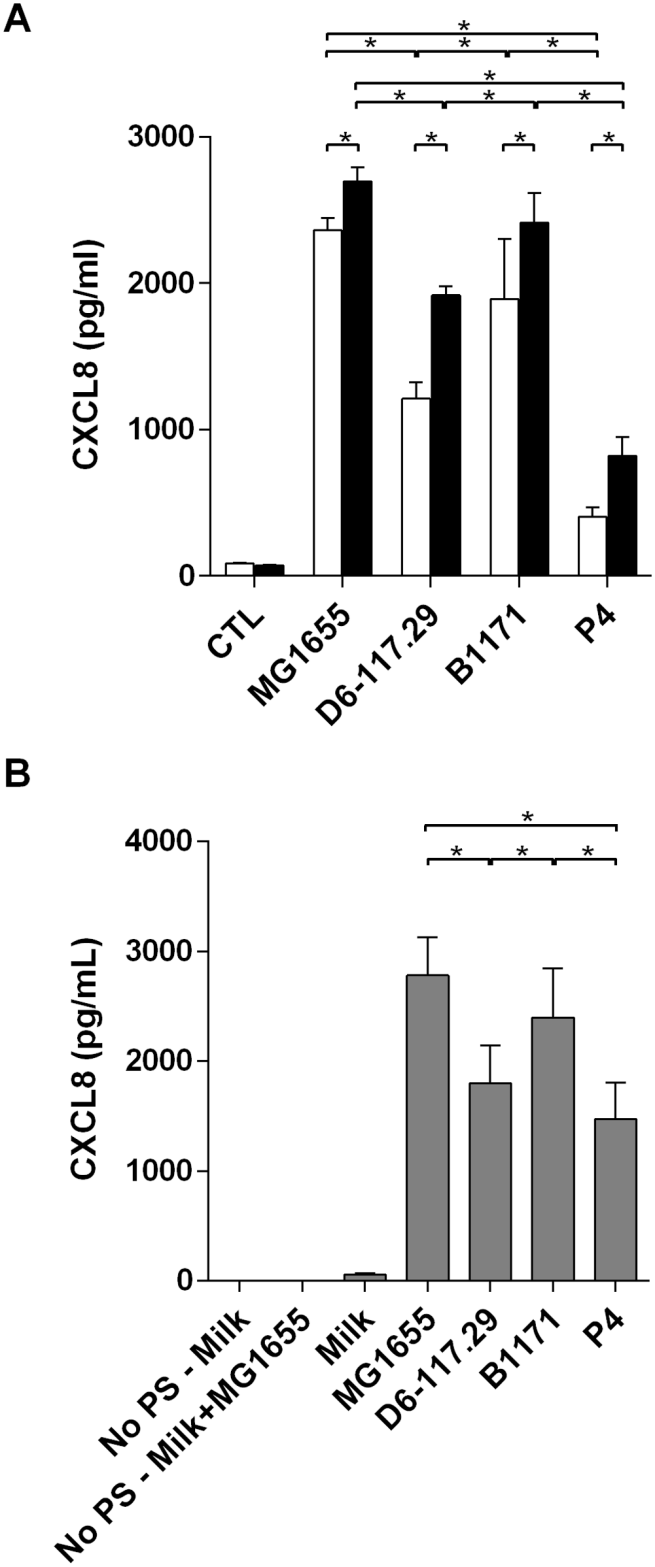
CXCL8 secretion induced by stimulation of PS cells with live *E*. *coli* strains. PS cells were incubated for 3 hours with the indicated strains at a MOI of 1 in stimulation medium (A) or fresh milk (B). Experiments in stimulation medium were performed in the absence of rbCD14 (white bars) or in the presence of 0.5 μg/ml rbCD14 (black bars). Experiments in milk were done without the addition of rbCD14. Cells were then washed twice with HBSS and medium with gentamicin was added. Response was analysed by quantification of CXCL8 secretion by ELISA 24h after beginning of the experiment. Data presented are mean values and SD obtained from four (-CD14) or three (+ CD14) independent experiments with stimulations performed in duplicates. * indicates statistical significance (P < 0.05). p-values were calculated using exact permutation tests after global comparison using a Kruskal and Wallis test.

We then analyzed the pro-inflammatory response of PS cells triggered by these different strains. In the absence of serum, results clearly show that strains MG1655 and B1171 induce strong proinflammatory responses by PS cells as evidenced by high levels of CXCL8 secretion in the medium (Fig 5). On the contrary, stimulation by strain P4 was the lowest while secretion triggered by strain D6-117.29 was intermediate. Because our previous results had indicated that CD14 was important for the recognition of LPS by PS cells, we purified recombinant bovine CD14 (rbCD14) and used it in stimulation assays [26]. Secretion was slightly increased by rbCD14 but, nevertheless, the low stimulation observed with strain P4 was still observed. When stimulations were performed in fresh milk, a pattern similar to that in medium was observed, showing that strain P4 was also stimulating PS cells weaker than strain MG1655 (Fig 5B). As control experiments, we showed that cells potentially present in the milk were not responsible for the measured secretion of CXCL8 (bar “No PS—Milk+MG1655”).

**Fig 6:**
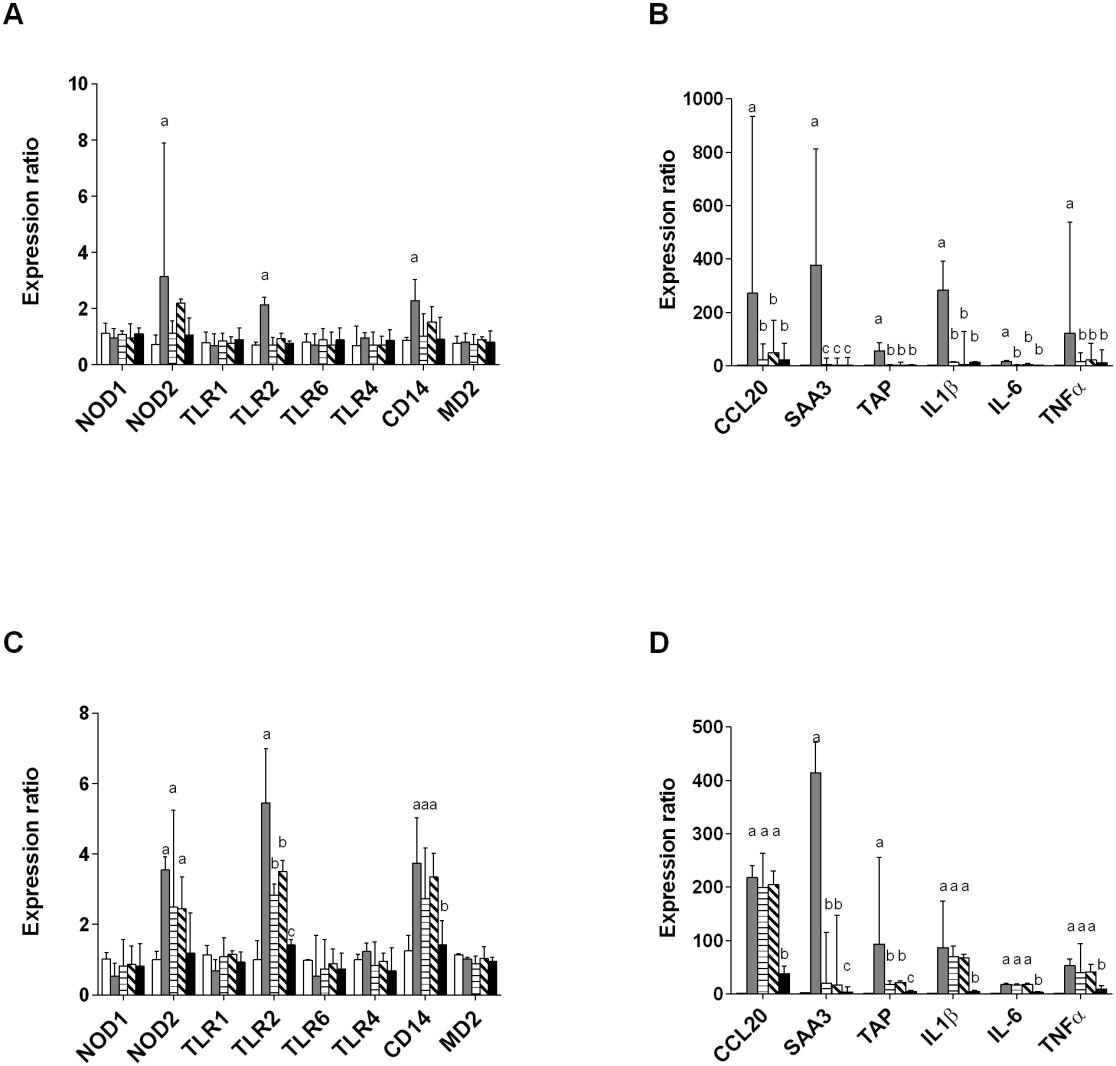
RT-qPCR analysis of the response of PS cells to stimulation by live *E*. *coli* strains. PS cells were incubated for 3 hours with the indicated strains at a MOI of 1 in stimulation medium without rbCD14. Cells were then washed twice with HBSS and medium with gentamicin was added. RNA was extracted 3 h p.i. (A-B) or 8 h p.i. (C-D). Response was analysed by RT-qPCR and values were normalized against expression of 18S, PPIA and BACT as described previously [31]. Strains used were: mock-infected (white bars), MG1655 (grey bars), D6-117.29 (horizontal stripes), B1171 (tilted stripes), P4 (black bars). Data presented are mean values and SD obtained from three independent experiments with stimulations performed in triplicates. For each gene studied, values with different letters are statistically different from the unstimulated cells and from each other (P < 0.05). p-values were calculated using exact permutation tests after global comparison using a Kruskal and Wallis test.

To get a more detailed view of the response of PS cells to *E*. *coli* strains, the expression of different genes controlling the expression of innate immunity receptors, cytokines, chemokines or defensins was also investigated (Fig 6). Regarding the expression of receptors (Fig 6A and 6C), an increased expression of NOD2, TLR2 and CD14 was observed at 3 h p.i. with strain MG1655 and with all strains except P4 at 8 h p.i.. No changes in the expression of NOD1, TLR1, TLR4, TLR6 and MD2 were detected.

Concerning effector genes, an increased expression was detected at 3 h p.i. and 8 h p.i. Again, the response triggered by strain P4 was much lower than that observed for the other strains. On the opposite, the response initiated by MG1655 was higher than that obtained with the other strains, in particular at 3 h p.i.. As a summary, strain P4 was the least potent in the stimulation of the innate response of PS cells while MG1655 triggered the highest responses.

### Repertoire of MAMPs expressed by *E*. *coli* strains

**Fig 7:**
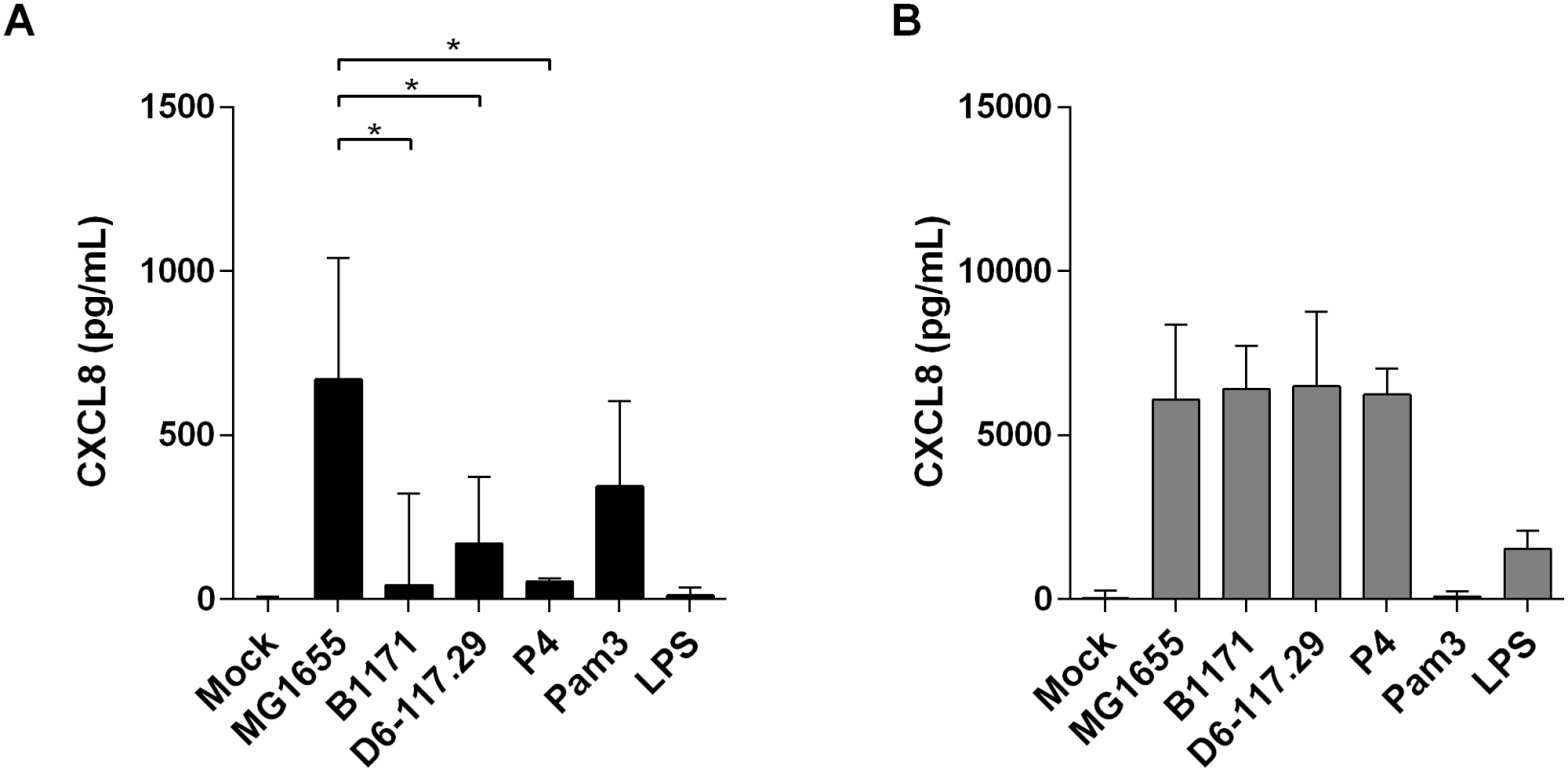
CXCL8 secretion by HEK-TLR2 (A) and HEK-TLR4(B) cells upon stimulation with live *E*. *coli* strains. Cells were incubated for 3 hours with the indicated strains at a MOI of 1. Cells were then delicately washed once with HBSS and medium with gentamicin was added. Response was analysed by quantification of CXCL8 secretion by ELISA 24h after beginning of the experiment. Data presented are mean values and SD obtained from three independent experiments with stimulations performed in duplicates. Values with different letters are statistically different from the mock-treated cells and from each other (P < 0.05). p-values were calculated using exact permutation tests after global comparison using a Kruskal and Wallis test.

In order to analyze if the differences in PS cell stimulation were the consequences of expression of different MAMP repertoires by the four *E*. *coli* strains, we used HEK cells expressing either TLR2 or TLR4. Response was analyzed in terms of CXCL8 production. Interestingly, while HEK/TLR4 cells responded in a similar fashion to stimulation with all four strains, HEK/TLR2 cells showed a pattern of response similar to PS cells with a higher response with MG1655 and lower responses with the other three strains suggesting different stimulation of the TLR2 pathway (Fig 7). These results suggested that agonists of PRRs produced by *E*. *coli* strains might differ quantitatively or qualitatively. However, these observations could not explain why P4 induced a lower response in bovine MECs.

### Sensitivity of *E*. *coli* to phagocytosis and bactericidal activity of bovine PMNs

**Fig 8:**
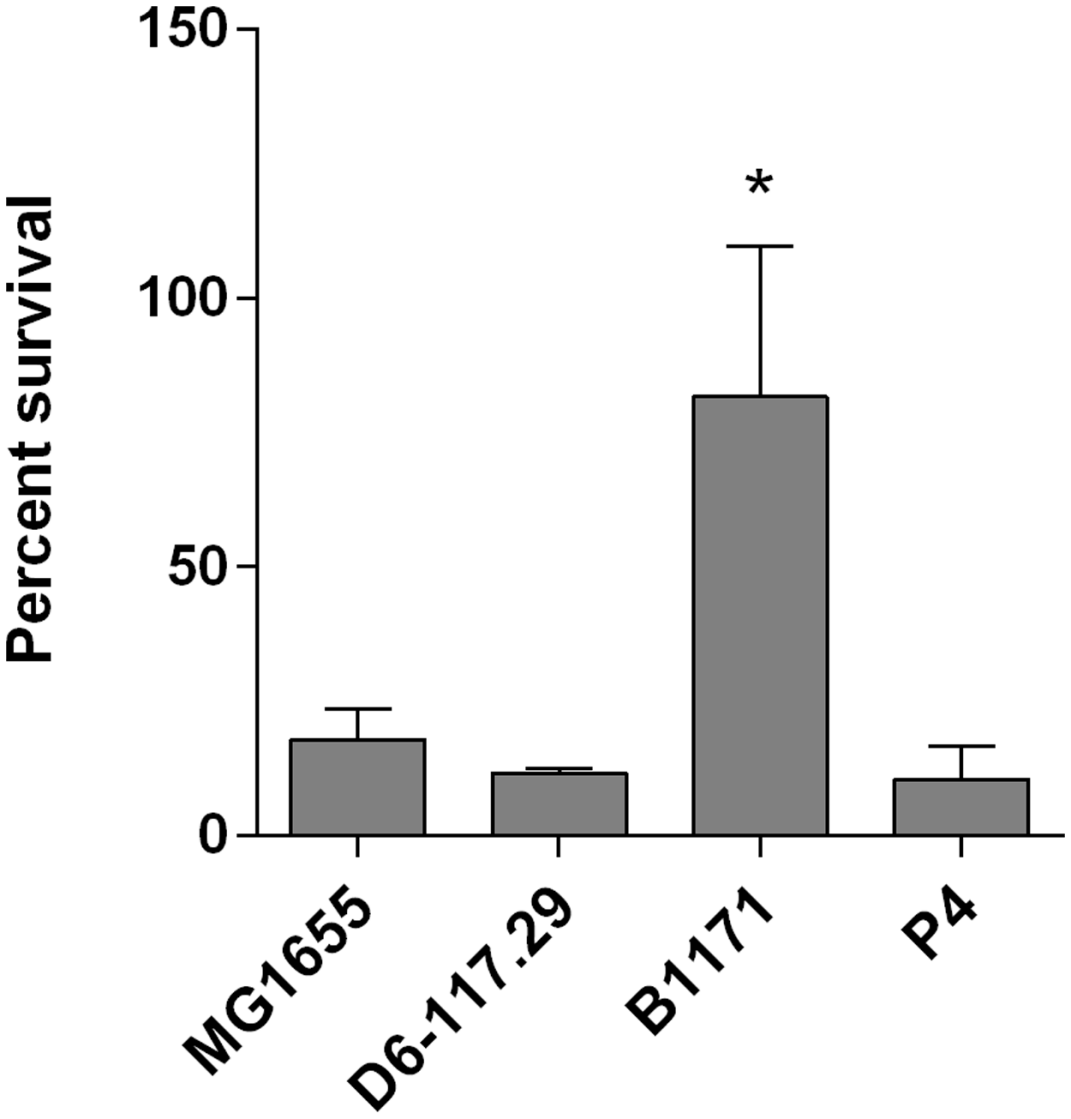
Resistance of *E*. *coli* strains to neutrophil killing. Bovine PMNs were incubated in the presence of 10% heat-inactivated serum with the indicated strains for one hour at a MOI of 10. Survival was calculated by dividing the number of bacteria recovered after incubation 1h in the presence of PMNs by the number of bacteria recovered after incubation 1h without any PMNs. Results are the mean and standard deviation from three independent experiments.

Once the innate immune response is triggered, neutrophils are massively recruited to the site of infection and are the main effectors that allow bacterial clearance. Another key determinant for the survival of strains to infection is therefore their ability to activate or to resist PMNs. Each strain was therefore incubated for 1 h in the presence of blood bovine PMNs (Fig 8). Only strain B1171 showed significant survival to neutrophils, while all the other strains were significantly killed by neutrophils (approx. 10–20% survival after 1 hour).

Cytospin followed by MGG staining clearly showed that B1171 bacteria were rarely found intracellular and neutrophils hardly showed any shape changes. On the opposite, the other strains were taken up by neutrophils with important morphological changes of neutrophils (data not shown). The failure of neutrophils to phagocytose strain B1171 is likely to explain its high survival to neutrophil killing.

## Discussion

The aim of the present report was to contribute to the identification of key phenotypes that contribute to the severity of mastitis.

Mice appeared as a useful *in vivo* model to evaluate the severity of infection induced by different *E*. *coli* strains. Indeed, comparable data were obtained in mice and cows using different strains [11]. The panel of strains tested was selected with the expectation that it would increase the diversity of responses to infection. We thus selected a well-known non-pathogenic *E*. *coli* strain, strain K-12 MG1655 [24]. We also selected the *E*. *coli* P4 strain used in a number of studies [32, 33]. Strain D6-117.29 was isolated from a case of severe clinical mastitis [9]. Strain B1171 was chosen from our strain collection based on its documented properties to resist neutrophil killing.

Results from mouse experiments showed that strains MG1655 and D6-117.29 were associated with non-severe mastitis characterized by low clinical scores, low bacterial loads in infected glands and reduced secretion of pro-inflammatory mediators. On the contrary, P4 and B1171 strains induced more severe mastitis with higher bacterial loads, higher neutrophil counts and higher secretion of IL-6 and MIP2, especially for strain P4.

The ability of strain MG1655 to trigger neutrophil recruitment, albeit of limited intensity, was rather unexpected since this strain is considered in the literature as a non-pathogenic strain. Yet, our data clearly indicate that it is able to stimulate a proinflammatory reaction from MECs which would explain the low inflammation observed *in vivo*. The absence of an O-antigen [34] could facilitate interactions between LPS from MG1655 and the TLR4 receptor, leading to a stronger stimulating action [35]. It could also allow for a better interaction between the TLR2 receptor and outer membrane motifs. This could explain the higher stimulation of HEK-TLR2 cells observed with strain MG1655. Despite this efficient stimulation of the innate immune system, the other properties of this strain, in particular its failure to grow in milk, its serum susceptibility and its sensitivity to neutrophil mediated killing, are likely to explain the low severity of infection it induces.

When it comes to the role of serum resistance in mastitis, some reports do not support a significant contribution to the development mastitis while others suggest a link with ability to trigger mastitis [1, 11, 36]. Nevertheless, our data suggest that serum resistance might contribute, to some extent, to the severity of infection. Indeed, the two strains inducing the most severe infections, P4 and B1171, are the most serum resistant strains of our panel. However, the level of serum resistance is not strictly linked to severity: additional experiments with a strain as resistant to serum as P4 induced clinical signs similar to those induced with the serum sensitive strain D6-117.29 (data not shown). A high level of resistance does not therefore imply severe infection but some level of serum resistance could be necessary to trigger mastitis.

Growth in milk is a pre-requisite for development of bacteria in the udder but it is not sufficient to trigger mastitis [37]. Indeed, although we observed different severity *in vivo*, growth rate in milk of all strains, except MG1655, were comparable. Compared to classical media (BHI for instance), growth rate was reduced in milk as had already been observed by others [37]. This might be due to the presence of various inhibitory components such as lactoferrin, complement factors, antibodies or proteins (caseins for example).

As PMNs are key cells of the resolution of mastitis, their viability and efficiency play a critical role in the pathogenesis of coliforms mastitis [38]. B1171 was the only strain almost fully resistant to phagocytosis by bovine PMNs. Strain P4 was as susceptible to neutrophil killing as the other strains. Interestingly, strain B1171 is actually an O8 strain that carries a gene cluster to express a group 4 capsule (located between *hisI* and *ugd*) and, in addition, shows a mucoid colony morphology (data not shown). The capsule of B1171 might thus contribute to the better resistance to phagocytosis, because of its weak agglutination by antibodies [39]. Our finding is reminiscent to previous reports showing that O8 strains had higher survival than other strains [40, 41].

Most interestingly, for the first time to our knowledge, the ability of different *E*. *coli* strains to stimulate mammary epithelial cells was compared to one another. These cells are important for the initiation of the innate immune response of the host as they have the ability to respond to a large range of bacterial components as well as to live bacteria [26, 28, 42]. We took advantage of a new mammary epithelial cell line to analyze how the different *E*. *coli* strains of this study stimulated the innate response [26].

Interestingly, the amplitude of response was not equal with all the strains: MG1655 and B1171 induced a stronger response than the other strains. Studies in terms of gene expression confirmed that strain MG1655 actually induced a higher response than all the other strains. Strikingly, P4 was the strain that stimulated MECs the least. This low response was observed both in the absence of CD14 and when CD14 was added to the medium indicating that it might not only be the presence of an O-antigen, which requires CD14 for signalling, that is responsible for this low stimulation.

These differences in activation of the innate immune system were not associated with differences in adhesion properties: adhesion to PS cells was similar for all strains. Whether invasion capacities could explain these different responses is not certain. Although strains P4 and D6-117.29 induce the lowest responses and have higher invasion abilities, the invasion rates are still very low (below 0.01%).

As an alternative explanation, it is possible that the repertoire of MAMPs produced by these different strains is responsible for the different pro-inflammatory responses. Our data obtained with HEK-TLR2 cells indeed suggest that strain MG1655 expresses more TLR2 agonists than the other three strains, or that TLR2 agonists are more readily accessible to the TLR2 receptor complex. Conversely, response of HEK-TLR4 cells did not unravel significant differences between strains.

*In vivo*, the mastitis induced by P4 shows a strong production of proinflammatory cytokines and chemokines, while this strain had a low stimulating power on MECs. An interesting hypothesis is therefore that this strain could divert the sensing capacity of the MECs, which would delay the triggering of the immune response of the udder. As a consequence, when the innate response is triggered, the inoculum has reached a significant level and the infection is more severe. To confirm this hypothesis, the genetic determinants responsible for the weak stimulation induced by P4 will need to be identified.

Altogether, the diversity of strain phenotypes observed in this report argues for multiple strategies that could be used by *E*. *coli* to cause mastitis. MG1655, B1171 and D6-117.29 are well recognized by MECs, and thus would induce a prompt and efficient immune response of the udder, contrary to P4. Yet, MG1655 and D6-117.29 being sensitive to milk complement factors, would be rapidly eliminated. The ability of P4 to resist certain components like milk complement would allow it to multiply in the udder without triggering any major inflammation in the first steps of colonization. When reaching a threshold of bacterial load, P4 would trigger an inflammation proportional to the bacterial load, thus recruiting massive amount of leukocytes. The peculiar case of B1171 would be attributed to its great resistance to PMNs and possibly by the presence in its genome of the *estA1* gene encoding a heat-stable enterotoxin (unpublished data) that could be responsible for mammary tissue lesions as described by others [43].

To conclude, our results, combined with recent genomic and phenotypic studies performed by us and others [7, 44, 45], suggest that properties of *E*. *coli* strains able to cause mastitis are highly variable from one strain to another, explaining the difficulty to define a specific serotype or pathotype for *E*. *coli* mastitis.

## References

[1] Hogan J, Larry Smith K. Coliform mastitis. Vet Res. 2003;34(5):507–19. Epub 2003/10/15. 10.1051/vetres:2003022 14556693

[2] Burvenich C, Van Merris V, Mehrzad J, Diez-Fraile A, Duchateau L. Severity of *E*. *coli* mastitis is mainly determined by cow factors. Vet Res. 2003;34(5):521–64. Epub 2003/10/15. 10.1051/vetres:2003023 14556694

[3] Schukken YH, Gunther J, Fitzpatrick J, Fontaine MC, Goetze L, Holst O, et al Host-response patterns of intramammary infections in dairy cows. Vet Immunol Immunopathol. 2011;144(3–4):270–89. Epub 2011/10/01. S0165-2427(11)00352-7 [pii] 10.1016/j.vetimm.2011.08.022 . 21955443

[4] Dogan B, Klaessig S, Rishniw M, Almeida RA, Oliver SP, Simpson K, et al Adherent and invasive *Escherichia coli* are associated with persistent bovine mastitis. Vet Microbiol. 2006;116(4):270–82. Epub 2006/06/22. 10.1016/j.vetmic.2006.04.023 16787715

[5] Nemeth J, Muckle CA, Gyles CL. In vitro comparison of bovine mastitis and fecal *Escherichia coli* isolates. Vet Microbiol. 1994;40(3–4):231–8. Epub 1994/06/01. . 7941288 10.1016/0378-1135(94)90112-0

[6] Blum SE, Leitner G. Genotyping and virulence factors assessment of bovine mastitis *Escherichia coli*. Vet Microbiol. 2013;163(3–4):305–12. Epub 2013/02/05. 10.1016/j.vetmic.2012.12.037 . 23374653

[7] Richards VP, Lefebure T, Pavinski Bitar PD, Dogan B, Simpson KW, Schukken YH, et al Genome based phylogeny and comparative genomic analysis of intra-mammary pathogenic *Escherichia coli*. PLoS One. 2015;10(3):e0119799 Epub 2015/03/26. 10.1371/journal.pone.0119799 ; 25807497 PMC4373696

[8] Blum SE, Heller ED, Sela S, Elad D, Edery N, Leitner G. Genomic and Phenomic Study of Mammary Pathogenic *Escherichia coli*. PLoS One. 2015;10(9):e0136387 Epub 2015/09/04. 10.1371/journal.pone.0136387 . 26327312 PMC4556653

[9] Kempf F, Slugocki C, Blum SE, Leitner G, Germon P. Genomic Comparative Study of Bovine Mastitis *Escherichia coli*. PLoS One. 2016;11(1):e0147954 10.1371/journal.pone.0147954 ; 26809117 PMC4725725

[10] Blum S, Heller ED, Krifucks O, Sela S, Hammer-Muntz O, Leitner G. Identification of a bovine mastitis *Escherichia coli* subset. Vet Microbiol. 2008;132(1–2):135–48. Epub 2008/06/24. 10.1016/j.vetmic.2008.05.012 18571344

[11] Anderson JC, Burrows MR, Bramley AJ. Bacterial adherence in mastitis caused by *Escherichia coli*. Vet Pathol. 1977;14(6):618–28. Epub 1977/11/01. 10.1177/030098587701400608 337635

[12] Dopfer D, Nederbragt H, Almeida RA, Gaastra W. Studies about the mechanism of internalization by mammary epithelial cells of *Escherichia coli* isolated from persistent bovine mastitis. Vet Microbiol. 2001;80(3):285–96. Epub 2001/05/05. . 11337144 10.1016/s0378-1135(01)00307-8

[13] Hill AW. Factors influencing the outcome of *Escherichia coli* mastitis in the dairy cow. Res Vet Sci. 1981;31(1):107–12. Epub 1981/07/01. . 7031811

[14] Heyneman R, Burvenich C, Vercauteren R. Interaction between the respiratory burst activity of neutrophil leukocytes and experimentally induced *Escherichia coli* mastitis in cows. J Dairy Sci. 1990;73(4):985–94. Epub 1990/04/01. 10.3168/jds.S0022-0302(90)78756-5 . 2161024

[15] Vandeputte-Van Messom G, Burvenich C, Roets E, Massart-Leen AM, Heyneman R, Kremer WD, et al Classification of newly calved cows into moderate and severe responders to experimentally induced Escherichia coli mastitis. J Dairy Res. 1993;60(1):19–29. Epub 1993/02/01. . 8436664 10.1017/s002202990002731x

[16] Elazar S, Gonen E, Livneh-Kol A, Rosenshine I, Shpigel NY. Essential role of neutrophils but not mammary alveolar macrophages in a murine model of acute *Escherichia coli* mastitis. Vet Res. 2010;41(4):53 Epub 2010/04/27. 10.1051/vetres/2010025 v100002 [pii] ; 20416261 PMC2881416

[17] Elazar S, Gonen E, Livneh-Kol A, Rosenshine I, Shpigel NY. Neutrophil recruitment in endotoxin-induced murine mastitis is strictly dependent on mammary alveolar macrophages. Vet Res. 2010;41(1):10 Epub 2009/10/16. 10.1051/vetres/2009058 v09453 [pii] ; 19828114 PMC2775169

[18] Akira S, Takeda K. Toll-like receptor signalling. Nat Rev Immunol. 2004;4(7):499–511. Epub 2004/07/02. 10.1038/nri1391 . 15229469

[19] Gunther J, Koy M, Berthold A, Schuberth HJ, Seyfert HM. Comparison of the pathogen species-specific immune response in udder derived cell types and their models. Vet Res. 2016;47:22 10.1186/s13567-016-0307-3 ; 26830914 PMC4736154

[20] Tattoli I, Travassos LH, Carneiro LA, Magalhaes JG, Girardin SE. The Nodosome: Nod1 and Nod2 control bacterial infections and inflammation. Semin Immunopathol. 2007;29(3):289–301. Epub 2007/08/11. 10.1007/s00281-007-0083-2 . 17690884

[21] Notebaert S, Meyer E. Mouse models to study the pathogenesis and control of bovine mastitis. A review. Vet Q. 2006;28(1):2–13. Epub 2006/04/12. 10.1080/01652176.2006.9695201 . 16605156

[22] Chandler RL. Experimental bacterial mastitis in the mouse. J Med Microbiol. 1970;3(2):273–82. Epub 1970/05/01. 10.1099/00222615-3-2-273 . 4989101

[23] Bramley AJ. Variations in the susceptibility of lactating and non-lactating bovine udders to infection when infused with *Escherichia coli*. J Dairy Res. 1976;43(2):205–11. Epub 1976/06/01. . 783219 10.1017/s0022029900015752

[24] Riley M, Abe T, Arnaud MB, Berlyn MK, Blattner FR, Chaudhuri RR, et al *Escherichia coli* K-12: a cooperatively developed annotation snapshot—2005. Nucleic Acids Res. 2006;34(1):1–9. 10.1093/nar/gkj405 16397293 PMC1325200

[25] Porcherie A, Gilbert FB, Germon P, Cunha P, Trotereau A, Rossignol C, et al IL-17A Is an Important Effector of the Immune Response of the Mammary Gland to Escherichia coli Infection. J Immunol. 2016;196(2):803–12. 10.4049/jimmunol.1500705 . 26685206

[26] Roussel P, Cunha P, Porcherie A, Petzl W, Gilbert FB, Riollet C, et al Investigating the contribution of IL-17A and IL-17F to the host response during Escherichia coli mastitis. Vet Res. 2015;46:56 10.1186/s13567-015-0201-4 ; 26062913 PMC4462179

[27] Rainard P, Riollet C, Berthon P, Cunha P, Fromageau A, Rossignol C, et al The chemokine CXCL3 is responsible for the constitutive chemotactic activity of bovine milk for neutrophils. Mol Immunol. 2008;45(15):4020–7. Epub 2008/07/29. S0161-5890(08)00234-4 [pii] 10.1016/j.molimm.2008.06.010 . 18657861

[28] Porcherie A, Cunha P, Trotereau A, Roussel P, Gilbert FB, Rainard P, et al Repertoire of *Escherichia coli* agonists sensed by innate immunity receptors of the bovine udder and mammary epithelial cells. Vet Res. 2012;43(1):14 Epub 2012/02/15. 10.1186/1297-9716-43-14 ; 22330199 PMC3305352

[29] Iwaki T, Castellino FJ. A single plasmid transfection that offers a significant advantage associated with puromycin selection in Drosophila Schneider S2 cells expressing heterologous proteins. Cytotechnology. 2008;57(1):45–9. 10.1007/s10616-008-9129-0 ; 19003171 PMC2553637

[30] Rainard P. The complement in milk and defense of the bovine mammary gland against infections. Vet Res. 2003;34(5):647–70. Epub 2003/10/15. 10.1051/vetres:2003025 . 14556699

[31] Bougarn S, Cunha P, Gilbert FB, Meurens F, Rainard P. Technical note: Validation of candidate reference genes for normalization of quantitative PCR in bovine mammary epithelial cells responding to inflammatory stimuli. J Dairy Sci. 2011;94(5):2425–30. Epub 2011/04/29. S0022-0302(11)00220-7 [pii] 10.3168/jds.2010-3859 [doi] . 21524534

[32] Riollet C, Rainard P, Poutrel B. Kinetics of cells and cytokines during immune-mediated inflammation in the mammary gland of cows systemically immunized with *Staphylococcus aureus* alpha-toxin. Inflamm Res. 2000;49(9):486–96. Epub 2000/11/09. 10.1007/s000110050621 11071124

[33] Vangroenweghe F, Rainard P, Paape M, Duchateau L, Burvenich C. Increase of *Escherichia coli* inoculum doses induces faster innate immune response in primiparous cows. J Dairy Sci. 2004;87(12):4132–44. Epub 2004/11/17. S0022-0302(04)73556-0 [pii] 10.3168/jds.S0022-0302(04)73556-0 . 15545375

[34] Stevenson G, Neal B, Liu D, Hobbs M, Packer NH, Batley M, et al Structure of the O antigen of *Escherichia coli* K-12 and the sequence of its rfb gene cluster. J Bacteriol. 1994;176(13):4144–56. Epub 1994/07/01. ; 7517391 10.1128/jb.176.13.4144-4156.1994 PMC205614

[35] Pupo E, Lindner B, Brade H, Schromm AB. Intact rough- and smooth-form lipopolysaccharides from *Escherichia coli* separated by preparative gel electrophoresis exhibit differential biologic activity in human macrophages. FEBS J. 2013;280(4):1095–111. Epub 2013/01/03. 10.1111/febs.12104 . 23279861

[36] Carroll EJ, Jain NC, Schalm OW, Lasmanis J. Experimentally induced coliform mastitis: inoculation of udders with serum-sensitive and serum-resistant organisms. Am J Vet Res. 1973;34(9):1143–6. Epub 1973/09/01. . 4583644

[37] Kornalijnslijper JE, van Werven T, Daemen AJ, van den Broek J, Niewold TA, Rutten VP, et al In vitro growth of mastitis-inducing *Escherichia coli* in milk and milk fractions of dairy cows. Vet Microbiol. 2003;91(2–3):125–34. Epub 2002/11/30. . 12458162 10.1016/s0378-1135(02)00266-3

[38] Mehrzad J, Duchateau L, Burvenich C. Viability of milk neutrophils and severity of bovine coliform mastitis. J Dairy Sci. 2004;87(12):4150–62. Epub 2004/11/17. 10.3168/jds.S0022-0302(04)73558-4 . 15545377

[39] Schembri MA, Dalsgaard D, Klemm P. Capsule shields the function of short bacterial adhesins. J Bacteriol. 2004;186(5):1249–57. Epub 2004/02/20. ; 10.1128/JB.186.5.1249-1257.2004 14973035 PMC344426

[40] Hill AW, Heneghan DJ, Williams MR. The opsonic activity of bovine milk whey for the phagocytosis and killing by neutrophils of encapsulated and non-encapsulated *Escherichia coli*. Vet Microbiol. 1983;8(3):293–300. Epub 1983/06/01. . 6351417 10.1016/0378-1135(83)90081-0

[41] Orskov I, Orskov F, Jann B, Jann K. Serology, chemistry, and genetics of O and K antigens of *Escherichia coli*. Bacteriol Rev. 1977;41(3):667–710. Epub 1977/09/01. ; 334154 10.1128/br.41.3.667-710.1977 PMC414020

[42] Gunther J, Koczan D, Yang W, Nurnberg G, Repsilber D, Schuberth HJ, et al Assessment of the immune capacity of mammary epithelial cells: comparison with mammary tissue after challenge with *Escherichia coli*. Vet Res. 2009;40(4):31 Epub 2009/03/27. 10.1051/vetres/2009014 v08328 [pii] ; 19321125 PMC2695127

[43] Brooker BE, Frost AJ, Hill AW. At least two toxins are involved in *Escherichia coli* mastitis. Experientia. 1981;37(3):290–2. Epub 1981/03/15. . 7016575 10.1007/BF01991661

[44] Goldstone RJ, Harris S, Smith DG. Genomic content typifying a prevalent clade of bovine mastitis-associated Escherichia coli. Scientific reports. 2016;6:30115 10.1038/srep30115 ; 27436046 PMC4951805

[45] Kempf F, Loux V, Germon P. Genome Sequences of Two Bovine Mastitis-Causing *Escherichia coli* Strains. Genome announcements. 2015;3(2). Epub 2015/04/11. 10.1128/genomeA.00259-15 25858841 PMC4392153

